# The Influence of Process Parameters on the Microstructural Properties of Spray-Pyrolyzed β-Ga_2_O_3_

**DOI:** 10.3390/nano13091455

**Published:** 2023-04-25

**Authors:** Constance Schmidt, Axel Fechner, Oleksandr Selyshchev, Dietrich R. T. Zahn

**Affiliations:** Semiconductor Physics, Chemnitz University of Technology, D-09107 Chemnitz, Germany

**Keywords:** β-Ga_2_O_3_, spray pyrolysis, spray coating, ultrasonic nebulization, microstructures, Raman spectroscopy, X-ray diffraction, scanning electron microscopy, residual stress measurements

## Abstract

In this work, the deposition of β-Ga_2_O_3_ microstructures and thin films was performed with Ga(NO_3_)_3_ solutions by ultrasonic nebulization and spray coating as low-cost techniques. By changing the deposition parameters, the shape of β-Ga_2_O_3_ microstructures was controlled. Micro-spheres were obtained by ultrasonic nebulization. Micro-flakes and vortices were fabricated by spray coating aqueous concentrated and diluted precursor solutions, respectively. Roundish flakes were achieved from water–ethanol mixtures, which were rolled up into tubes by increasing the number of deposition cycles. Increasing the ethanol-to-water ratio allows continuous thin films at an optimal Ga(NO_3_)_3_ concentration of 0.15 M and a substrate temperature of 190 °C to be formed. The monoclinic β-Ga_2_O_3_ phase was achieved by thermal annealing at 1000 °C in an ambient atmosphere. Scanning electronic microscopy (SEM), X-ray diffraction (XRD), and UV-Raman spectroscopy were employed to characterize these microstructures. In the XRD study, in addition to the phase information, the residual stress values were determined using the sin^2^(ψ) method. Raman spectroscopy confirms that the β-Ga_2_O_3_ phase and relative shifts of the Raman modes of the different microstructures can partially be assigned to residual stress. The high-frequency Raman modes proved to be more sensitive to shifting and broadening than the low-frequency Raman modes.

## 1. Introduction

Due to its wide bandgap of around 4.9 eV and n-type conductivity [1], β-Ga_2_O_3_ is a promising transparent oxide semiconductor (TOS) for applications in the field of transparent transistors [2,3], solar cells [4,5], and other optoelectronic devices [6,7,8]. Gallium oxide forms five polymorphs (α, β, γ, δ, and ε) that are different in terms of stability, optical bandgap, and crystal lattice. Among them, monoclinic β-Ga_2_O_3_ is the most stable phase [9]. Due to its thermal stability, high dielectric constant, and high breakdown voltage, β-Ga_2_O_3_ is also considered an emerging material for high-power electronics [1].

The techniques established for obtaining the β-Ga_2_O_3_ layers include, for instance, chemical vapor deposition [10], plasma-enhanced chemical vapor deposition [11,12], electron beam deposition [7], molecular beam epitaxy [13], pulsed laser deposition [14], radio-frequency magnetron sputtering [15], sol–gel [16], and hydro- and solvothermal methods [17,18,19]. However, low-cost techniques, such as ultrasonic nebulization and spray coating, are only represented in a few studies [20,21,22,23,24,25,26,27,28,29,30,31,32]. The latest reviews about β-Ga_2_O_3_ rarely consider spray pyrolysis as a deposition method [1,33,34]. In contrast to many other deposition techniques, spray pyrolysis does not require vacuum conditions and has the advantages of a simple and low-cost technique for obtaining thin films and micro-/nanostructures. In the case of gallium oxide, spray pyrolysis can be performed in an air atmosphere, making it potentially scalable.

Since morphology is a vital factor for the physical and chemical properties of a material, gallium oxides attract interest beyond closed films. Ga_2_O_3_ structures, such as nanowires and nanobelts, are already actively investigated [35,36,37,38,39,40]. Additionally, Ga_2_O_3_ spheres produced by an aerosol-spray method gained attention [21,25]. Quasi-two-dimensional (2D) β-Ga_2_O_3_ flakes were reported by us recently [41]. The progress in spray pyrolysis allows for a variety of morphologies to be obtained, depending on the process parameters [42], which are also determined by the precursors, solvents, and additives used. In addition, the choice of the generator with its carrier gas, as well as the heat source for the spray pyrolysis process, are important.

Here, we study the influence of the process parameters used in spray pyrolysis on the microstructural properties of β-Ga_2_O_3_ in detail by employing a low-cost ultrasonic nebulizer and spray coater setups. We found five different structures, which range from three-dimensional spheres to closed films. The samples were investigated using SEM to obtain the morphology of the microstructures, XRD to determine the crystalline phase, the crystallite size, and residual stress, and Raman spectroscopy for additional information on crystalline phase and lattice dynamics.

## 2. Materials and Methods

In order to prepare β-Ga_2_O_3_ microstructures, gallium nitrate Ga(NO_3_)_3_ Puratronic^®^, 99.999% (Alfa Aesar, Karlsruhe, Germany) was used as a precursor. Ga(NO_3_)_3_ was dissolved in water (H_2_O) or water–ethanol (H_2_O/EtOH) mixtures. The Ga(NO_3_)_3_ concentration was varied between 4.5 × 10^−3^ M and 0.3 M. Optionally, a tenside soap solution was used as an additive to change the surface morphology of β-Ga_2_O_3_ spheres.

**Figure 1:**
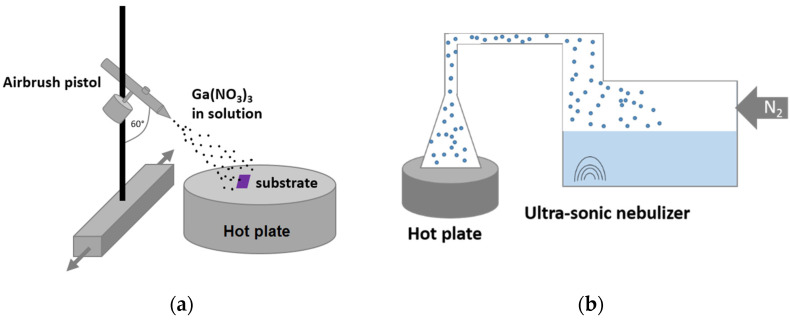
Schematics of the deposition setups: (**a**) spray coating setup based on an airbrush pistol, which is mounted on a linear stage; (**b**) ultrasonic nebulizer setup. N_2_ was used in both cases as a carrier gas.

Spray pyrolysis was performed using home-built spray coating and ultrasonic nebulization setups, which are sketched in Figure 1. The spray coating setup (Figure 1a) consists of an airbrush pistol mounted at an angle of around 60° with respect to the substrate, a linear motorized stage, and a hot plate. Pressurized N_2_ was used as carrier gas. A fine mist of gallium nitrate solution was sprayed onto a heated Si substrate. In order to obtain homogenous coverage, the airbrush pistol was moved by a motorized stage, typically using ten runs equal to 65 s of spray coating. The ultrasonic nebulizer setup (Figure 1b) consists of a household air humidifier, a pipe system, and a hot plate. The fine mist of gallium nitrate solution, produced in the nebulizer, was directed to a substrate via a pipe using a N_2_ flow as a carrier. During deposition, the Si(111) substrates were heated above 110 °C, which is the decomposition temperature of Ga(NO_3_)_3_. Deposition temperature and time were varied. For obtaining β-phase gallium oxide, the as-deposited amorphous samples were annealed at 1000 °C for 5 min in an air atmosphere. Samples were loaded in a muffle oven heated to the predetermined temperature. After annealing was completed, samples were removed from the oven and cooled naturally.

For the investigation of the morphology of the samples, a scanning electronic microscope (Nova NanoSEM (FEI Company, Hillsboro, OR, USA) with a Schottky field emission gun) was used with voltages of 15 to 20 kV applied. Measurements were conducted in immersion mode. Raman spectroscopy was performed using a LabRam HR 800 setup (HORIBA Jobin Yvon GmbH, Bensheim, Germany). The Raman signal was collected in the backscattering geometry and dispersed by a grating of 2400 lines/mm. A 325 nm He-Cd laser was used for excitation with a laser power of 4.5 mW. The standard accumulation time was 30 s and was repeated 10 times. The XRD measurements were carried out using a SmartLab X-ray diffractometer (Rigaku Corporation, Tokyo, Japan) with a wavelength of 1.5406 Å and a power of 45 kW. Samples were measured with a parallel beam geometry. We used θ–2θ scans for the phase analysis in a range from 15° to 70° and a speed of 3°/min. For this measurement, a Ge-monochromator was used. In addition, residual stress measurements were performed using the sin^2^(ψ) method. The 2θ angle chosen for this measurement was 64.7°. The sample tilt ψ was varied between 0° and 45° with a constant sin^2^(ψ) distance. To increase the signal, residual stress measurements were performed without the usage of a monochromator. To determine the stress, we used the following equation [43,44,45]:

(1) $$\sigma =\frac{E}{d_{0}(1+\nu )}\cdot \frac{∆d}{∆\sin2(\psi )}$$

where *σ* is the stress, *E* is Young’s modulus, *d*_0_ is the unstrained lattice parameter, *d* is the lattice parameter with strain, *ν* is Poisson’s ratio, and Δ*d/*Δsin^2^(*ψ*) is the slope determined from the plot of *d* vs. sin^2^(ψ). Values for *E* and *ν* were taken from [46]. The equation combines Hook’s law and Bragg’s law, using the strain in the sample by tilting it by the angle ψ. The sheet resistance was measured by a 4-point probe method using a Keithley 2636A source meter (Cleveland, OH, USA).

## 3. Results and Discussion

### 3.1. Scanning Electronic Microscopy (SEM)

**Figure 2:**
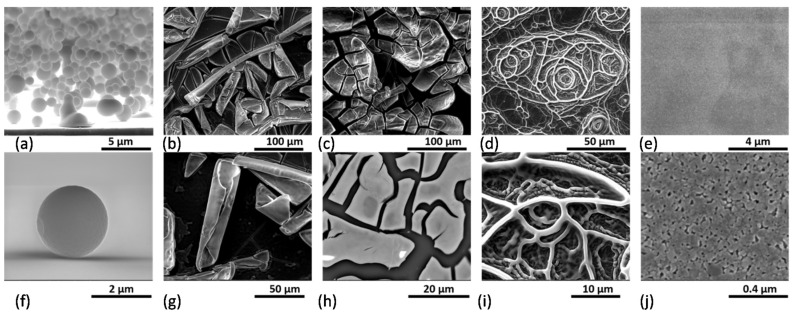
SEM images of different β-Ga_2_O_3_ structures: (**a**,**f**) spheres, (**b**,**g**) tubes, (**c**) roundish flakes and (**h**) flat flakes, (**d**,**i**) vortices, and (**e**,**j**) thin films.

The morphology of the prepared β-Ga_2_O_3_ microstructures was investigated by SEM. By varying the deposition parameters, we were able to achieve five different morphologies summarized in Table 1 and shown in Figure 2.

Spherical structures were the only ones we observed in the ultrasonic nebulization (USN) deposition of β-Ga_2_O_3_ over a broad range of gallium nitrate precursor concentrations, types of solvent, and substrate temperature ranges. A typical example of β-Ga_2_O_3_ spheres prepared by the USN is shown in Figure 2a,f. The spheres were formed using water, as well as water–ethanol solvent mixtures. Changing the deposition temperature from 120 °C to 230 °C made no difference in the formation of the Ga_2_O_3_ spheres. The concentration of Ga(NO_3_)_3_ only influenced the size of the spheres. Such behavior can be explained by the fact that the mist droplets produced by USN take a sphere-like shape. Since the deposition temperature was set higher than the boiling points of the solvents, we can assume that the solvents at least partially evaporate before reaching the substrate. This was sufficient for the gallium oxide particles to take the sphere-like droplet shape.

By changing the deposition parameters, it was possible to control the uniformity of these microstructures. Figure S1a–c show β-Ga_2_O_3_ spheres with different surface roughness. Introducing a surfactant to aqueous Ga(NO_3_)_3_ led to a significantly increased surface roughness since the surface tension gets lower. Adding ethanol to the Ga(NO_3_)_3_ aqueous solution reduced the roughness of the sphere surfaces. It was also possible to control the size of the spheres by changing the concentration used. A higher concentration of Ga(NO_3_)_3_ led to larger spheres. By continuing the deposition for a long time, e.g., for 4 h, we were able to stack the β-Ga_2_O_3_ spheres on each other (see Figure 2a). Tubes of β-Ga_2_O_3_ were prepared using the spray coating (SC) method. Typical SEM images of the tubes are shown in Figure 2b,g. The deposition was performed in several iterations. First, a 0.17 M Ga(NO_3_)_3_ solution in H_2_O and EtOH (3:1 volume ratio) was spray-coated at a deposition temperature of 170 °C followed by annealing at 1000 °C for 5 min. After cooling, a fresh portion of Ga(NO_3_)_3_ solution was sprayed, and the sample was annealed again. In total, the procedure was repeated three times. The use of the water–ethanol mixture was crucial for obtaining the tubes. By adding ethanol to the Ga(NO_3_)_3_ water solution, we obtained roundish flakes of β-Ga_2_O_3_ in the first iteration. In this regard, we can assume that the further deposition of the gallium nitrate precursor does not grow new Ga_2_O_3_ flakes of similar shape but builds up more oxide on the existing flakes, thus changing their morphology. After three iterations, rolled-up tubes were formed.

Flakes of β-Ga_2_O_3_ were obtained by spray coating using Ga(NO_3_)_3_ solutions either in H_2_O or H_2_O/EtOH as the solvents. These β-Ga_2_O_3_ microstructures are represented in Figure 2c,h. The deposition was performed in one iteration. Apparently, a change in concentration revealed only a minor influence on the morphology. Flakes were formed in a concentration range of 0.25–0.35 M Ga(NO_3_)_3_. The deposition temperature varied between 120 °C and 170 °C and had a minor effect on the morphology, while the change from water to a water–ethanol mixture had a stronger influence. Adding EtOH to the precursor solution causes the flakes to start rolling up from the edges. This may be due to the faster evaporation of the water–ethanol solvent mixture and the high concentration of Ga(NO_3_)_3_. The film dried quickly and curved up so that flat and roundish flakes of Ga_2_O_3_ remained.

Vortices of β-Ga_2_O_3_ were formed at very low Ga(NO_3_)_3_ concentration and increased deposition temperature. Their typical images are shown in Figure 2d,i. The deposition was performed in one iteration using 4.5 mM of aqueous Ga(NO_3_)_3_ solution. The deposition temperature varied from 210 °C to 230 °C. Because of the low concentration of the material and the high deposition temperature, the solution dries immediately on the substrate surface, resulting in the vortices of flower-like morphology. An elongated shape of the microstructures may appear due to the spray pattern of the airbrush pistol. Since the pistol was attached at an angle with respect to the substrate, we obtain an elliptical rather than a circular spray pattern.

Thin films of β-Ga_2_O_3_ were obtained at an intermediate concentration of 0.15 M Ga(NO_3_)_3_ in ethanol–water mixtures at a deposition temperature of 190 °C. The typical overview and zoomed images of the films can be seen in Figure 2e,j, respectively. Optimal concentration, deposition temperature, and adding the EtOH to the solution were crucial for the preparation of closed thin films. Higher and lower deposition temperatures with the same solution led to cracks in the film’s surface.

To optimize the quality of spray-coated β-Ga_2_O_3_ thin films, we investigated the influence of the EtOH:H_2_O volume ratio. Changing the EtOH:H_2_O ratio from 1:2 to 7:1 led to an increase in the film homogeneity, as can be seen in Figure S2a–h. While the pore size of the thin films generally decreased with higher EtOH content, a ratio of 2:1 was out of this trend. The pores for this sample were bigger. So far, we have no explanation for this behavior. In addition to the homogeneity, the film thickness changed. Using the same preparation parameters, the film thickness (determined by SEM cross-section) decreased from ≈320 nm for the films prepared with the 1:2 ratio to ≈140 nm for the films prepared with a ratio of 7:1. The reason for this thickness decrease is most likely the faster evaporation of EtOH in comparison to the ones used with a 1:2 or 1:1 ratio due to the lower boiling point of EtOH (78.4 °C) compared to H_2_O (100.0 °C).

**Scheme 1:**
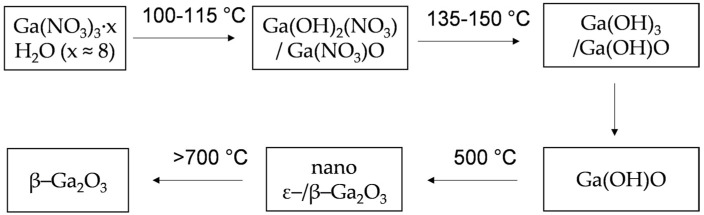
Pyrolysis process of Ga(NO_3_)_3_ ·xH_2_O and the formation of β-Ga_2_O_3_ [47,48].

From a chemistry point of view, Ga_2_O_3_ is formed by the pyrolysis of gallium nitrate (see Scheme 1). According to thermogravimetric studies by Berbenni et al., at 100–115 °C, crystallohydrate Ga(NO_3_)_3_·xH_2_O (x ≈ 8) loses water and decomposes, resulting in Ga(OH)_2_(NO_3_) [47]. At 135–150 °C, nitrogen is completely eliminated, resulting in Ga(OH)_3_ and Ga(OH)O. Nominally, Ga_2_O_3_ forms only at ≥500 °C. According to Playford et al., products of such calcination at 500 °C are a mixture of nanocrystalline hexagonal ε-Ga_2_O_3_ and β-Ga_2_O_3_ [48]. The pure phase of monoclinic β-Ga_2_O_3_ forms above 700 °C. Since ethanol evaporates faster than water, we assume that adding ethanol does not significantly change the chemistry of Ga(NO_3_)_3_ decomposition. The role of ethanol can be understood as facilitating the evaporation of droplets, which determines the shape of the nebulized spheres and rolls up the spray-coated flakes and tubes. At high concentrations of ethanol, it increases the affinity of Ga(NO_3_)_3_ solutions to the hydrophobic silicon surface and allows a homogeneous distribution of the material on the surface forming the films.

In view of the interest in the β-Ga_2_O_3_ for electrical applications [8], we tested the resistance of the prepared films and microstructures. The closed films on silicon substrates showed dielectric behavior with sheet resistance higher than 1 GΩ/square. It is known that depending on the growth conditions, e.g., inert or oxygen atmosphere, doping, type of substrate, etc., both dielectric and n-type conductive Ga_2_O_3_ can be achieved [49,50,51]. In our case, the high sheet resistance is likely due to the β-Ga_2_O_3_ films prepared in air and without doping, e.g., by group IV elements [50,51]. Another reason for the high resistance can be the polycrystalline nature of the microstructures and the absence of preferred orientation of β-Ga_2_O_3_ on the Si(111) substrate. The sheet resistance of unclosed films of spheres, tubes, and vortices was in the range of MΩ/square. The much smaller resistance was not due to Ga_2_O_3_ itself, but rather to the semiconducting silicon substrate accessible to the electrical probes. In terms of applications, Ga_2_O_3_ thin films on silicon could be proposed, for example as dielectric gate material in metal-oxide semiconductors (MOS) [52] or for simultaneous passivation and doping of silicon wafers [12]. Ga_2_O_3_ microstructures other than films could be of interest for gas sensors or catalytic applications [1,40,53,54].

### 3.2. X-ray Diffraction (XRD)

**Figure 3:**
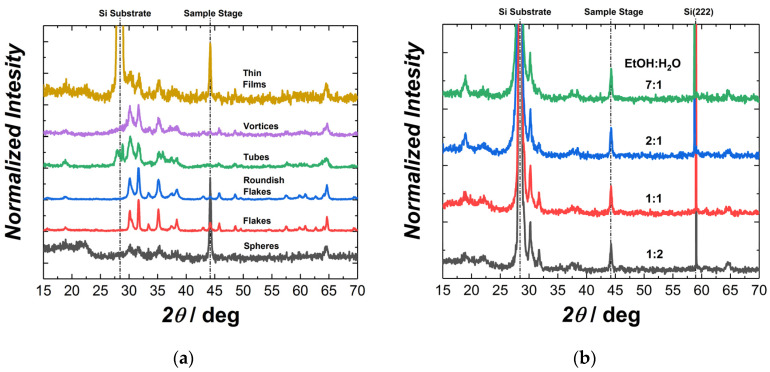
XRD patterns (**a**) for all β-Ga_2_O_3_ microstructures normalized to the reflection at 31.7° of the (002) plane and (**b**) for β-Ga_2_O_3_ thin films with different EtOH:H_2_O ratios normalized to the reflection at 30.2° corresponding to the (−110) plane.

To investigate the crystal phase and crystallite size of the different gallium oxide microstructures, an XRD study was performed. The XRD scans of all β-Ga_2_O_3_ microstructures are shown in Figure 3a. For all annealed samples, the XRD patterns show reflections corresponding to the literature pattern of β-Ga_2_O_3_ (ICSD #83645). The assignment of the XRD peaks is given in the Supplementary part (Figure S3). For instance, an intensive reflection at around 31.7° corresponds to the (002) lattice plane. The highest reflection at 64.7°, which still gives a significant signal, is due to the (512) lattice plane. This peak was used for residual stress measurements for all microstructures, as discussed later. Since several lattice planes of β-Ga_2_O_3_ are visible, the samples are polycrystalline. The signal-to-noise ratio cannot be used as an indication of crystallinity here, since samples had different thicknesses. The β-gallium oxide phase was expected because the annealing temperature of the samples at 1000 °C was higher than the known transition temperature (800 °C) from α- to β-Ga_2_O_3_ [55]. The β-phase of gallium oxide is monoclinic with the lattice parameters of a = 12.23 Å, b = 3.04 Å, c = 5.80 Å, α = γ = 90°, and β = 103.8° [9]. In the case of thin films, in addition to reflections of β-Ga_2_O_3_, the Si substrate was visible at 28.4°, and sometimes the steel sample holder by a reflection at 44.3° was registered. At the same time, no reflections from other gallium oxide polymorphs were detected. For example, the most intense reflection of α-Ga_2_O_3_ (ICSD #27431), expected to be at 33.8°, was not observed here.

The crystallite sizes for all microstructures were calculated using Debye–Scherrer’s equation [56]. The crystallite size gradually increases from 13 nm to 33 nm with increasing Ga(NO_3_)_3_ concentration from 0.15 M to 0.35 M. Interestingly, the calculated crystal size followed a linear behavior with a 9.5 nm increase for every 0.1 M (see Figure S4a). However, β-Ga_2_O_3_ vortices were out of this trend. Despite the fact that vortices were prepared with a very low concentration of 4.5 mM, they show a relatively large crystallite size. By now, we have no explanation for this behavior. We can only assume that it is related to a higher deposition temperature used for vortexes compared to other morphologies. A higher deposition temperature can result in a larger initial crystallite grain size, which subsequently increases during annealing.

The XRD scans for thin films obtained with a different solvent ratio are shown in Figure 3b. Compared to the XRD of the microstructures shown in Figure 3a, one can see that the orientation of the thin films changed, with the most intense peak now at 30.2°. While the 31.7° peak belongs to the (002) plane, the 30.2° reflection represents the (−110) lattice planes. Additionally, due to the thin film thickness, we can clearly see the Si(111) substrate peak at 29°, the forbidden Si(222) peak at 59°, and the sample holder at ~44°. Similar to the other microstructures, thin films represent polycrystalline β-Ga_2_O_3_. Due to the low sample thickness, the scans are relatively noisy. The intensity of the Si substrate peak increased with increasing EtOH content. This result confirms the decrease in thickness when ethanol is added to the aqueous Ga(NO_3_)_3_ solution. The crystallite size for the thin films estimated using the Debye–Scherrer equation was on average (15 ± 3) nm (see Figure S4b).

### 3.3. Raman Spectroscopy

Raman spectroscopy was performed to verify the β-modification of Ga_2_O_3_ and to reveal changes in the vibrational properties among the different microstructures. Since the crystal structure of β-Ga_2_O_3_ consists of Ga_2_O_6_ octahedra and GaO_4_ tetrahedra, we can separate the Raman spectra into three main parts: the libration and translation of the chains (100–300 cm^−1^), the deformation of GaO_6_ octahedra (300–550 cm^−1^), and the stretching and bending of GaO_4_ tetrahedra (550–800 cm^−1^) [57]. The crystal structure, the separation of the Raman spectrum of β-Ga_2_O_3_, and the origin of all Raman active modes, are given in the Supplementary part (Table S1, Figures S5 and S6).

**Figure 4:**
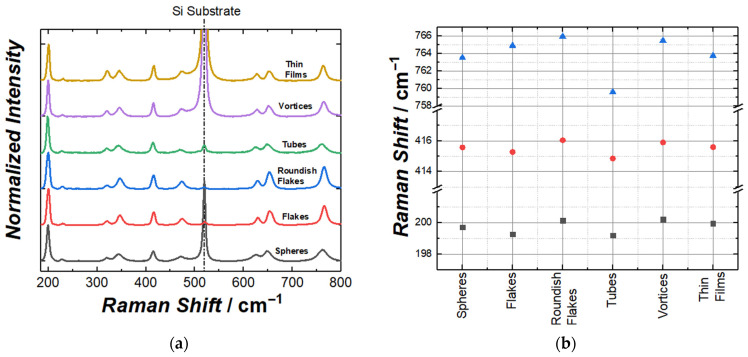
(**a**) Raman spectra for different β-Ga_2_O_3_ microstructures, normalized to the most intensive mode of β-Ga_2_O_3_, which is around 200 cm^−1^; (**b**) Raman mode positions, one for every part of the Raman spectrum of β-Ga_2_O_3_, in dependence of the microstructures.

Figure 4a shows the experimental Raman spectra for all microstructures obtained. Note that the peak at 320 cm^−1^ stems from the CaF_2_ objective used for UV-Raman spectra. This peak overlays with Raman modes of β-Ga_2_O_3_. In every case, the β-modification of Ga_2_O_3_ was confirmed by the Raman spectra. Even though the spectra were similar, a shift of certain modes was observed. The most pronounced changes in the Raman mode position in each of the three parts of the Raman spectrum of different β-Ga_2_O_3_ microstructures are summarized in Figure 4b. It is obvious that the microstructure type influences the shift of the β-Ga_2_O_3_ modes. A possible cause of these shifts may be the stress in the microstructures. The stress persists in the samples due to the “shock” process parameters used in the spray pyrolysis and subsequent processing (relatively low deposition temperature, rapid crystallization from the solution or its vapor, rapid annealing). To determine the stress values, residual stress measurements with XRD were performed and discussed below.

Even though all microstructures show the fingerprints of β-Ga_2_O_3_, small differences between the spectra can be noticed. First of all, the Si substrate peak at 520 cm^−1^ is more pronounced for samples with a low layer thickness (thin films). Furthermore, from the spectra normalized to the most intense peak of β-Ga_2_O_3_ at 200 cm^−1^, we can see that the intensity ratios between the features of different microstructures change. Reasons for this intensity ratio change are the structure itself, less crystallinity, and the fact that β-Ga_2_O_3_ is an anisotropic material. For example, it is known that the Raman mode intensities change for single crystalline β-Ga_2_O_3_ depending on the polarization geometry [58]. Additionally, curved or rounded structures, such as tubes and spheres, have a larger full width at half maximum (FWHM), while the other microstructures have lower FWHMs. Of all microstructures, thin films have the lowest FWHM. A comparison between microstructure and Raman FWHM can be found in the Supplementary part (Figure S7).

**Figure 5:**
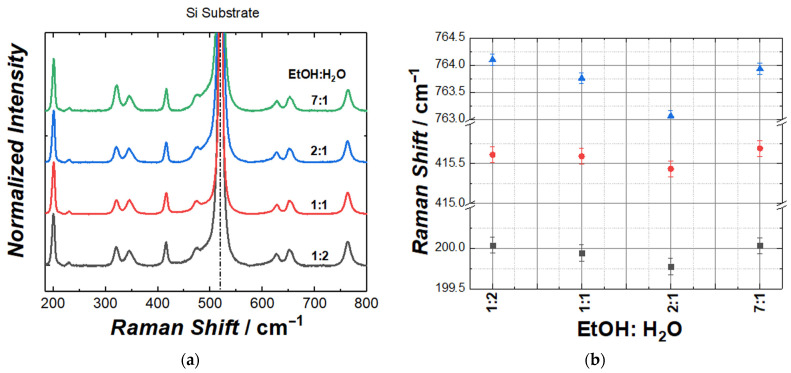
Raman spectroscopy of thin β-Ga_2_O_3_ films with different EtOH:H_2_O ratios: (**a**) spectra; (**b**) mode position in dependence of the EtOH:H_2_O ratio.

Additionally, we studied Raman spectra of the β-Ga_2_O_3_ thin films prepared at different solvent ratios as in Figure 5a. Because the films are thin (≈320 nm and less), the strong Si peak at 520 cm^−1^ from the substrate dominated the spectra. The Raman mode of the CaF_2_ objective at 320 cm^−1^ is also typically observed. The other peaks observed correspond to the β-Ga_2_O_3_ phase. The Raman spectra for these thin films are very similar. Figure 5b represents the mode position in dependence on the EtOH:H_2_O ratio. The sample prepared with the 2:1 ratio revealed a marked redshift of the Raman modes, which was more pronounced for higher frequency modes. Judging by the SEM images, the ratio of 2:1 led to bigger pores in the film. Due to this slightly different morphology, the Raman shifts may be caused by stress. It should be noted that the differences between the films themselves are less significant than between the films and other microstructures. The FWHMs of the Raman modes for these films hardly change between the different EtOH:H_2_O ratios. For instance, the mode around 200 cm^−1^ only changes from 6.0 cm^−1^ to 6.1 cm^−1^, which is still in the range of the error bars (±0.1 cm^−1^). The most significant difference was between the 2:1 and 7:1 samples, where the FWHMs for the peak around 765 cm^−1^ were 11.9 cm^−1^ and 14.6 cm^−1^, respectively. This confirms our observation that the high-frequency modes are more sensitive to changes in the film quality. The high-frequency peaks in gallium oxides correspond to the stretching and bending modes of tetrahedra. In the tetrahedra, the distances between Ga and O atoms are shorter and the force constants are stronger. So, even a small change in the interatomic distance caused by strain makes these Raman modes more sensitive to shifts and broadening.

### 3.4. Residual Stress Measurements

**Figure 6:**
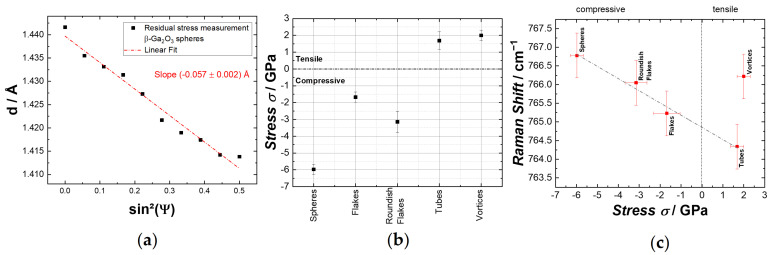
(**a**) sin^2^(ψ)-d-plot with a linear fit to determine the slope; (**b**) stress results for all microstructures of β-Ga_2_O_3_; (**c**) plot of Raman shift of all microstructures in dependence of the measured stress values for the mode around 765 cm^−1^. Similar plots for modes around 200 cm^−1^ and 415 cm^−1^ are shown in the Supplementary Material, Figure S8. The dotted line is a guideline showing a linear trend for Raman shifts with sign and stress values. Vortices do not fit this trend.

Residual stress measurements were performed using the sin^2^(ψ) method. Figure 6a shows a typical plot of this measurement using the spheres of β-Ga_2_O_3_ as an example. A linear behavior and a negative slope indicate compressive stress in this sample. Using Equation (1), we calculated the stress value in this film to be (−5.98 ± 0.31) GPa. Figure 6b and Table S2 summarize the stress values determined for all microstructures.

Most of the samples such as spheres, flakes, and thin films showed compressive stress, while vortices and tubes exhibited tensile stress. Significant stress in the samples can be understood, on one hand, because the morphology of the microstructures is far away from the prism shape typical for monoclinic crystals. On the other hand, deposition at relatively low temperatures and a short annealing time at high annealing temperatures are not enough to release residual stress.

Even though there is no clear trend of the stress values in relation to the process parameters of the prepared samples, several observations and assumptions can be made. Spheres experience the highest stress of all microstructures prepared. The flakes showed the most relaxed microstructure compared to the other samples, which may be due to their breaking apart at high-stress points during the deposition process. The rolling up of the edges of the flakes causes higher stress in the roundish flakes. Tubes show positive stress values that may be due to the longer annealing time due to deposition in several iterations. The reason for the stress can be the microstructures themselves. The small crystallites of β-Ga_2_O_3_ dry on the substrate in droplets and remain in this form. Staying in this formation requires large deformation in β-Ga_2_O_3_, which leads to high stress values.

Figure 6c and Figure S8 show the Raman frequencies of the microstructures plotted over the stress values. Theoretically, compressive stress reduces the distance between atoms and causes an upward shift of the Raman modes [59]. For instance, spheres, which reveal the highest compressive stress of all microstructures prepared, exhibit the highest positive Raman shift for the modes in all three regions. For tensile stress, the Raman shift should be the opposite. However, considering all samples, no clear dependence on stress is found. Therefore, thin films, roundish flakes, flakes, and partially tubes show Raman peaks in a similar range. The vortices are an exception. They exhibit Raman shifts toward higher values, while the tensile stress measured for this structure should cause a downward Raman shift. The observed contradictions may have two reasons. First, XRD and micro-Raman probes have dramatically different volumes. The X-ray beam is as large as several mm^2^, while micro-Raman measures microareas (µm^2^), determined by the laser focus. The macroscopic stress values obtained from the XRD do not necessarily have to be the same at the microlevel. Secondly, the crystal lattice of β-Ga_2_O_3_ is not as simple as cubic materials, such as silicon. The monoclinic lattice behaves differently under external pressure, e.g., compressibility values depend on the crystallographic direction and even the size of the particles (nano- or micro-powders) [60,61]. Since we deal here with a variety of microstructures, we can assume that the microstructure itself can also influence the Raman shift. If one excludes the vortices sample, a certain linear trend of the Raman modes with the internal stress can be found, which is most pronounced for the high-frequency mode at 765 cm^−1^ (see Figure 6c). For the low- and middle-frequency ranges at around 200 cm^−1^ and 415 cm^−1^, the trend is less obvious because of lower Raman shifts partially overlapping in the range of the measurement errors (Figure S8a,b).

These results confirm that the higher-frequency Raman modes are more sensitive to stress than the lower-frequency Raman modes. While the mode around 200 cm^−1^ only shifts in a range of (1.5 ± 0.6) cm^−1^, the mode around 765 cm^−1^ shifts in a range of (3.0 ± 0.6) cm^−1^. Even though the XRD-derived stress values are quite high (up to 6.0 ± 0.3 GPa), the shifts of the Raman modes are rather low. The results obtained qualitatively agree with the high-pressure studies on β-Ga_2_O_3_ [61,62]. The compressive stress produced by pressures below 21.6 GPa for β-Ga_2_O_3_ nanocrystals and below 22.2 GPa for microcrystals (without phase transition) shifts the Raman peaks in the positive direction. The phonon pressure coefficients were much larger for high-frequency Raman modes, e.g., 4–5 cm^−1^/GPa for the 764–766 cm^−1^ mode compared to low-frequency modes, e.g., 0.8–0.9 cm^−1^/GPa for the mode at 200 cm^−1^ [61,62]. The Grüneisen parameter, which represents the Raman shift with the relative volume change, for β-Ga_2_O_3_ differs a lot depending on the nano- or microcrystalline nature of the sample.

As for the thin films, the stress values were also measured using the sin^2^(ψ)-d-plot method. The residual stress vs. Raman shifts of β-Ga_2_O_3_ is shown in the Supplementary part (Figure S9). The general trend was that the compressive residual stress in the films was reduced with increasing ethanol concentration. However, similar to the observations made before, the sample with an EtOH:H_2_O ratio of 2:1 was an exception. The XRD-derived stress value was the highest in this sample. This correlates with the SEM images, showing larger pores for this sample. Samples with higher and lower ethanol content show smaller pore sizes, which generally decreased with an increasing ethanol/water ratio. Moreover, the stress in the thin film samples changed from significantly compressive to slightly tensile by adding more ethanol to the solution, while keeping the concentration of the precursor constant. No Raman shift–stress relationship was found (Figure S9). Since the films were prepared under the same conditions and exhibited a similar morphological pattern, small Raman shifts at significant internal stress may indicate that the films are stressed at the macrolevel probed by XRD and relaxed at the microlevel examined by micro-Raman.

## 4. Conclusions

In this work, we show the possibility to produce different β-Ga_2_O_3_ microstructures using low-cost spray pyrolysis techniques. These microstructures are flakes, tubes, vortices, thin films, and spheres. When changing deposition parameters, we can control the structure. Raman spectra indicate that the β-Ga_2_O_3_ phase is obtained for every structure. XRD measurements confirm the polycrystalline single phase of β-Ga_2_O_3_. The calculated crystallite size for β-Ga_2_O_3_ prepared by spray pyrolysis can be related to the Ga(NO_3_)_3_ concentration used.

Raman spectroscopy shows that all microstructures prepared are indeed β-Ga_2_O_3_. Additionally, even though the precursor was always Ga(NO_3_)_3_, Raman shifts of the specific modes are observable.

The residual stress measurements by the sin^2^(ψ) method show that the different microstructures of β-Ga_2_O_3_ have not only different stress but also change from compressive to tensile stress. Thin films prepared with different EtOH:H_2_O ratios reveal a change in stress for higher EtOH contents from compressive to tensile.

The stress in all samples investigated was compared with the Raman shifts. We can see some relation between the stress in the samples and the Raman shift. However, the Raman shift is not only influenced by stress, but also by the microstructure itself.

## Figures and Tables

**Table 1:** Process parameters for the deposition of different β-Ga_2_O_3_ structures. In every case, the annealing temperature was 1000 °C for 5 min.

	Spheres	Tubes	Flakes	Vortices	Films
**Technique**	USN	SC	SC	SC	SC
**Solvents for Ga(NO_3_)_3_**	H_2_O/ H_2_O + EtOH	H_2_O + EtOH (3:1)	H_2_O/ H_2_O + EtOH (3:1)	H_2_O	H_2_O:EtOH (2:x), x ≥ 1
***c*** **/M**	Independent	0.17	0.25–0.35	4.5 × 10^−3^	0.15
***T*** **_d_** **/°C**	120–230	170	120–170	210–230	190

USN—ultrasonic nebulization, SC—spray coating, *c*—the concentration of Ga(NO_3_)_3_ (M = mol/L) in water (H_2_O) or water and ethanol (H_2_O + EtOH) solvents, and *T*_d_—the deposition temperature.

## Data Availability

The data presented in this study are available upon request from the authors.

## Supplementary Materials

The following supporting information can be downloaded at https://www.mdpi.com/article/10.3390/nano13091455/s1, Figure S1: SEM images of differently shaped β-Ga2O3 spheres; Figure S2: SEM images of β-Ga2O3 thin films; Figure S3: Indexing of the XRD pattern of β-Ga2O3; Figure S4: Calculated crystallite sizes; Figure S5: Sketch of the crystal lattice of β-Ga2O3; Figure S6: Raman spectrum of bulk-like β-Ga2O3; Figure S7: FWHM of the Raman modes for different β-Ga2O3 microstructures; Figure S8: Raman shifts of all microstructures depend on the measured stress; Figure S9: Raman shifts of thin films depend on the measured stress; Table S1: An explanation for the origin of the Raman modes; Table S2: Stress and Raman shift for the different microstructures. References [41,57,63] are cited in the Supplementary Materials.

## References

[1] Pearton S.J., Yang J., Cary P.H., Ren F., Kim J., Tadjer M.J., Mastro M.A. (2018). A review of Ga_2_O_3_ materials, processing, and devices. Appl. Phys. Rev..

[2] Higashiwaki M., Sasaki K., Kuramata A., Masui T., Yamakoshi S. (2012). Gallium oxide (Ga_2_O_3_) metal-semiconductor field-effect transistors on single-crystal β-Ga_2_O_3_ (010) substrates. Appl. Phys. Lett..

[3] Matsuzaki K., Hiramatsu H., Nomura K., Yanagi H., Kamiya T., Hirano M., Hosono H. (2006). Growth, structure and carrier transport properties of Ga_2_O_3_ epitaxial film examined for transparent field-effect transistor. Thin Solid Films.

[4] Minami T., Nishi Y., Miyata T. (2013). High-efficiency Cu_2_O-based heterojunction solar cells fabricated using a Ga_2_O_3_ thin film as n-type layer. Appl. Phys. Express.

[5] Ueda N., Hosono H., Waseda R., Kawazoe H. (1997). Synthesis and control of conductivity of ultraviolet transmitting β-Ga_2_O_3_ single crystals. Appl. Phys. Lett..

[6] Tomm Y., Ko J., Yoshikawa A., Fukuda T. (2001). Floating zone growth of β-Ga_2_O_3_: A new window material for optoelectronic device applications. Sol. Energy Mater. Sol. Cells.

[7] Passlack M., Schubert E.F., Hobson W.S., Hong M., Moriya N., Chu S.N.G., Konstadinidis K., Mannaerts J.P., Schnoes M.L., Zydzik G.J. (1995). Ga_2_O_3_ films for electronic and optoelectronic applications. J. Appl. Phys..

[8] Galazka Z. (2018). β-Ga_2_O_3_ for wide-bandgap electronics and optoelectronics. Semicond. Sci. Technol..

[9] Geller S. (1960). Crystal Structure of β-Ga_2_O_3_. J. Chem. Phys..

[10] Choi Y.C., Kim W.S., Park Y.S., Lee S.M., Bae D.J., Lee Y.H., Park G.-S., Choi W., Lee N.S., Kim J.M. (2000). Catalytic Growth of β-Ga_2_O_3_ Nanowires by Arc Discharge. Adv. Mater..

[11] Wu C., Guo D.Y., Zhang L.Y., Li P.G., Zhang F.B., Tan C.K., Wang S.L., Liu A.P., Wu F.M., Tang W.H. (2020). Systematic investigation of the growth kinetics of *β*-Ga_2_O_3_ epilayer by plasma enhanced chemical vapor deposition. Appl. Phys. Lett..

[12] Allen T.G., Ernst M., Samundsett C., Cuevas A. Demonstration of c-Si solar cells with gallium oxide surface passivation and laser-doped gallium p+ regions. Proceedings of the 2015 IEEE 42nd Photovoltaic Specialist Conference, PVSC 2015.

[13] Sasaki K., Kuramata A., Masui T., Víllora E.G., Shimamura K., Yamakoshi S. (2012). Device-quality β-Ga_2_O_3_ epitaxial films fabricated by ozone molecular beam epitaxy. Appl. Phys. Express.

[14] Orita M., Ohta H., Hirano M., Hosono H. (2000). Deep-ultraviolet transparent conductive β-Ga_2_O_3_ thin films. Appl. Phys. Lett..

[15] Park S.Y., Lee S.Y., Seo S.H., Noh D.Y., Kang H.C. (2013). Self-catalytic growth of β-Ga_2_O_3_ nanowires deposited by radio-frequency magnetron sputtering. Appl. Phys. Express.

[16] Kim J.S., Kim H.E., Kwon A.K., Park H.L., Kim G.C. (2007). Effect of initial pH on nanophosphor β-Ga_2_O_3_:Eu^3+^ prepared through sol–gel process. J. Lumin..

[17] Guo D.Y., Chen K., Wang S.L., Wu F.M., Liu A.P., Li C.R., Li P.G., Tan C.K., Tang W.H. (2020). Self-Powered Solar-Blind Photodetectors Based on α/β Phase Junction of Ga_2_O_3_. Phys. Rev. Appl..

[18] He C., Guo D., Chen K., Wang S., Shen J., Zhao N., Liu A., Zheng Y., Li P., Wu Z. (2019). α-Ga_2_O_3_ Nanorod Array-Cu_2_O Microsphere p-n Junctions for Self-Powered Spectrum-Distinguishable Photodetectors. ACS Appl. Nano Mater..

[19] Sinha G., Patra A. (2009). Generation of green, red and white light from rare-earth doped Ga_2_O_3_ nanoparticles. Chem. Phys. Lett..

[20] Hao J., Cocivera M. (2002). Optical and luminescent properties of undoped and rare-earth-doped Ga_2_O_3_ thin films deposited by spray pyrolysis. J. Phys. D Appl. Phys..

[21] Kuai L., Wang J., Ming T., Fang C., Sun Z., Geng B., Wang J. (2015). Aerosol-spray diverse mesoporous metal oxides from metal nitrates. Sci. Rep..

[22] Kim H., Kim W. (1987). Optical properties of β-Ga_2_O_3_ and α-Ga_2_O_3_:Co thin films grown by spray pyrolysis. J. Appl. Phys..

[23] Ortiz A., Alonso J.C., Andrade E., Urbiola C. (2001). Structural and Optical Characteristics of Gallium Oxide Thin Films Deposited by Ultrasonic Spray Pyrolysis. J. Electrochem. Soc..

[24] Hao J., Lou Z., Renaud I., Cocivera M. (2004). Electroluminescence of europium-doped gallium oxide thin films. Thin Solid Films.

[25] Ogi T., Kaihatsu Y., Iskandar F., Tanabe E., Okuyama K. (2009). Synthesis of nanocrystalline GaN from Ga_2_O_3_ nanoparticles derived from salt-assisted spray pyrolysis. Adv. Powder Technol..

[26] Thomas S.R., Adamopoulos G., Lin Y.-H., Faber H., Sygellou L., Stratakis E., Pliatsikas N., Patsalas P.A., Anthopoulos T.D. (2014). High electron mobility thin-film transistors based on Ga_2_O_3_ grown by atmospheric ultrasonic spray pyrolysis at low temperatures. Appl. Phys. Lett..

[27] Winkler N., Wibowo R.A., Kautek W., Ligorio G., List-Kratochvil E.J.W., Dimopoulos T. (2019). Nanocrystalline Ga_2_O_3_ films deposited by spray pyrolysis from water-based solutions on glass and TCO substrates. J. Mater. Chem. C.

[28] Abejide F.H., Ajayi A.A., Akinsola S.I., Alabi A.B. (2022). Properties of gallium oxide thin film prepared on silicon substrate by spray pyrolysis method. J. Mater. Sci..

[29] Panov D.I., Zhang X., Spiridonov V.A., Azina L.V., Nuryev R.K., Prasolov N.D., Sokura L.A., Bauman D.A., Bougrov V.E., Romanov A.E. (2022). Thin films of gallium oxide obtained by spray-pyrolysis: Method and properties. Mater. Phys. Mech..

[30] Raphael R., Anila E. (2021). Investigation of photoluminescence emission from β-Ga_2_O_3_: Ce thin films deposited by spray pyrolysis technique. J. Alloys Compd..

[31] Raphael R., Devasia S., Shaji S., Anila E. (2022). Effect of substrate temperature on the properties of spray deposited Ga_2_O_3_ thin films, for solar blind UV detector applications. Opt. Mater..

[32] Schmidt C., Zahn D.R.T. (2022). Effect of impurities on the Raman spectra of spray-coated β-Ga_2_O_3_ thin films. J. Vac. Sci. Technol. A Vac. Surf. Film..

[33] Cooke J., Sensale-Rodriguez B., Ghadbeigi L. (2021). Methods for synthesizing β-Ga_2_O_3_ thin films beyond epitaxy. J. Phys. Photonics.

[34] Higashiwaki M., Sasaki K., Murakami H., Kumagai Y., Koukitu A., Kuramata A., Masui T., Yamakoshi S. (2016). Recent progress in Ga_2_O_3_ power devices. Semicond. Sci. Technol..

[35] Dai C., Tan H., Geng H. (2002). Model for assessing the melting on Hugoniots of metals: Al, Pb, Cu, Mo, Fe, and U. J. Appl. Phys..

[36] Zhang J., Jiang F., Zhang L. (2004). Fabrication, structural characterization and optical properties of semiconducting gallium oxide nanobelts. Phys. Lett. A.

[37] Liang C.H., Meng G.W., Wang G.Z., Wang Y.W., Zhang L.D., Zhang S.Y. (2001). Catalytic synthesis and photoluminescence of β-Ga_2_O_3_ nanowires. Appl. Phys. Lett..

[38] Kuo C.-L., Huang M.H. (2008). The growth of ultralong and highly blue luminescent gallium oxide nanowires and nanobelts, and direct horizontal nanowire growth on substrates. Nanotechnology.

[39] Makeswaran N., Kelly J.P., Haslam J.J., McKeown J.T., Ross M.S., Ramana C.V. (2022). Crystallization, Phase Stability, Microstructure, and Chemical Bonding in Ga_2_O_3_ Nanofibers Made by Electrospinning. ACS Omega.

[40] Tien L.-C., Chen W.-T., Ho C.-H. (2011). Enhanced Photocatalytic Activity in β-Ga_2_O_3_ Nanobelts. J. Am. Ceram. Soc..

[41] Schmidt C., Rahaman M., Zahn D.R.T. (2022). Conversion of 2-dimensional GaSe to 2-dimensional β-Ga_2_O_3_ by thermal oxidation. Nanotechnology.

[42] Jung D.S., Bin Park S., Kang Y.C. (2010). Design of particles by spray pyrolysis and recent progress in its application. Korean J. Chem. Eng..

[43] Cullity B.D., Stock S.R. (2001). Elements of X-ray Diffraction.

[44] Noyan I.C., Cohen J.B. (1987). Residual Stress: Measurement by Diffraction and Interpretation.

[45] Fitzpatrick M.E., Fry A.T., Holdway P., Kandil F.A., Shackleton J., Suominen L. (2006). Measurement Good Practice Guide No. 52. Determination of Residual Stresses by X-ray Diffraction.

[46] Grashchenko A.S., Kukushkin S.A., Nikolaev V.I., Osipov A.V., Osipova E.V., Soshnikov I.P. (2018). Study of the Aniso-tropic Elastoplastic Properties of β-Ga_2_O_3_ Films Synthesized on SiC/Si Substrates. Phys. Solid State.

[47] Berbenni V., Milanese C., Bruni G., Marini A. (2005). Thermal decomposition of gallium nitrate hydrate Ga(NO_3_)_3_×xH_2_O. J. Therm. Anal. Calorim..

[48] Playford H.Y., Hannon A.C., Barney E.R., Walton R.I. (2013). Structures of Uncharacterised Polymorphs of Gallium Oxide from Total Neutron Diffraction. Chem.—Eur. J..

[49] Nakano Y., Jimbo T. (2003). Interface properties of thermally oxidized *n*-GaN metal–oxide–semiconductor capacitors. Appl. Phys. Lett..

[50] Paskaleva A., Spassov D., Terziyska P. (2017). Electric, dielectric and optical properties of Ga_2_O_3_ grown by metal organic chemical vapour deposition. J. Phys. Conf. Ser..

[51] Gogova D., Schmidbauer M., Kwasniewski A. (2015). Homo- and heteroepitaxial growth of Sn-doped β-Ga_2_O_3_ layers by MOVPE. Crystengcomm.

[52] Kaya A., Mao H., Gao J., Chopdekar R.V., Takamura Y., Chowdhury S., Islam M.S. (2017). An Investigation of Electrical and Dielectric Parameters of Sol–Gel Process Enabled β-Ga_2_O_3_ as a Gate Dielectric Material. IEEE Trans. Electron Devices.

[53] Lampe U., Fleischer M., Meixner H. (1994). Lambda measurement with Ga_2_O_3_. Sens. Actuators B Chem..

[54] Shao T., Zhang P., Jin L., Li Z. (2013). Photocatalytic decomposition of perfluorooctanoic acid in pure water and sewage water by nanostructured gallium oxide. Appl. Catal. B.

[55] Meng Y., Gao Y., Chen K., Lu J., Xian F., Xu L., Zheng G., Kuang W., Cao Z. (2021). Annealing induced phase transition and optical properties of Ga_2_O_3_ thin films synthesized by sputtering technique. Optik.

[56] Scherrer P. (1918). Bestimmung der Größe und der inneren Struktur von Kolloidteilchen mittels Röntgenstrahlen. Nachr. Ges. Wiss..

[57] Dohy D., Lucazeau G., Revcolevschi A. (1982). Raman spectra and valence force field of single-crystalline β Ga_2_O_3_. J. Solid State Chem..

[58] Kranert C., Sturm C., Schmidt-Grund R., Grundmann M. (2016). Raman tensor elements of β-Ga_2_O_3_. Sci. Rep..

[59] De Wolf I. (1999). Stress measurements in Si microelectronics devices using Raman spectroscopy. J. Raman Spectrosc..

[60] Lipinska-Kalita K.E., Chen B., Kruger M.B., Ohki Y., Murowchick J., Gogol E.P. (2003). High-pressure X-ray diffraction studies of the nanostructured transparent vitroceramic medium K_2_O−SiO_2_−Ga_2_O_3_. Phys. Rev. B.

[61] Machon D., McMillan P.F., Xu B., Dong J. (2006). High-pressure study of the β-to-α transition in Ga_2_O_3_. Phys. Rev. B.

[62] Del Moral Cejudo A. (2017). High-Pressure Optical and Vibrational Properties of Ga2O3 Nanocrystals. https://diposit.ub.edu/dspace/handle/2445/123247.

[63] Momma K., Izumi F. (2011). VESTA 3 for three-dimensional visualization of crystal, volumetric and morphology data. J. Appl. Crystallogr..

