# Supplementary material for: The Influence of Process Parameters on the Microstructural Properties of Spray-Pyrolyzed β-Ga2O3
Source: Nanomaterials (Basel). 2023 Apr 25;13(9):1455. doi: 10.3390/nano13091455 (PMC10179802; doi:10.3390/nano13091455)
Supplement: Supplementary file 1 [file nanomaterials-13-01455-s001.zip › nanomaterials-2316372-supplementary.pdf]

Supplementary Materials

Influence of Process Parameters on the Microstructural Properties of Spray-Pyrolyzed β-Ga_2O_3

Constance Schmidt, Axel Fechner, Oleksandr Selyshchev, and Dietrich R. T. Zahn

Semiconductor Physics, Chemnitz University of Technology, D-09107 Chemnitz, Germany

*Correspondence: oleksandr.selyshchev@physik.tu-chemnitz.de, zahn@physik.tu-chemnitz.de

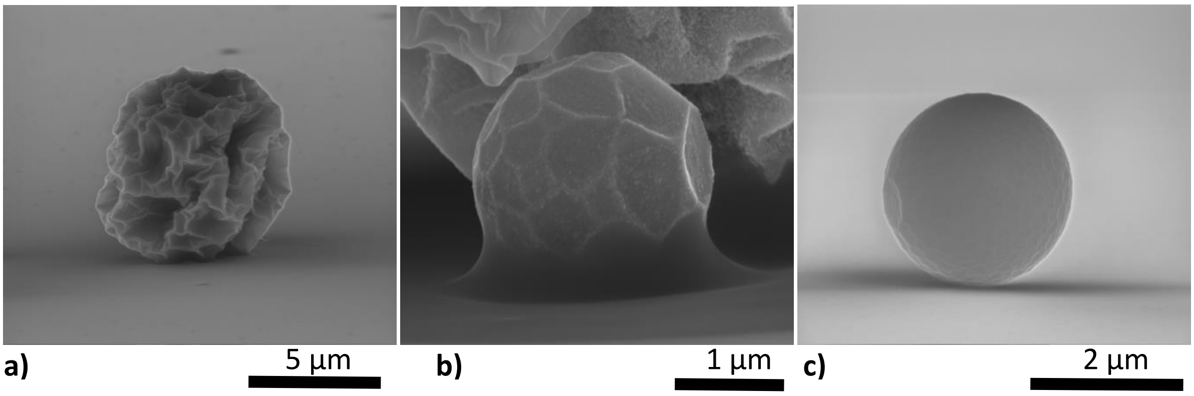

Figure S1. SEM images of differently shaped β-Ga_2O_3 spheres: a) by adding tenside solution; b) without any additions; and c) with adding ethanol to the solution.

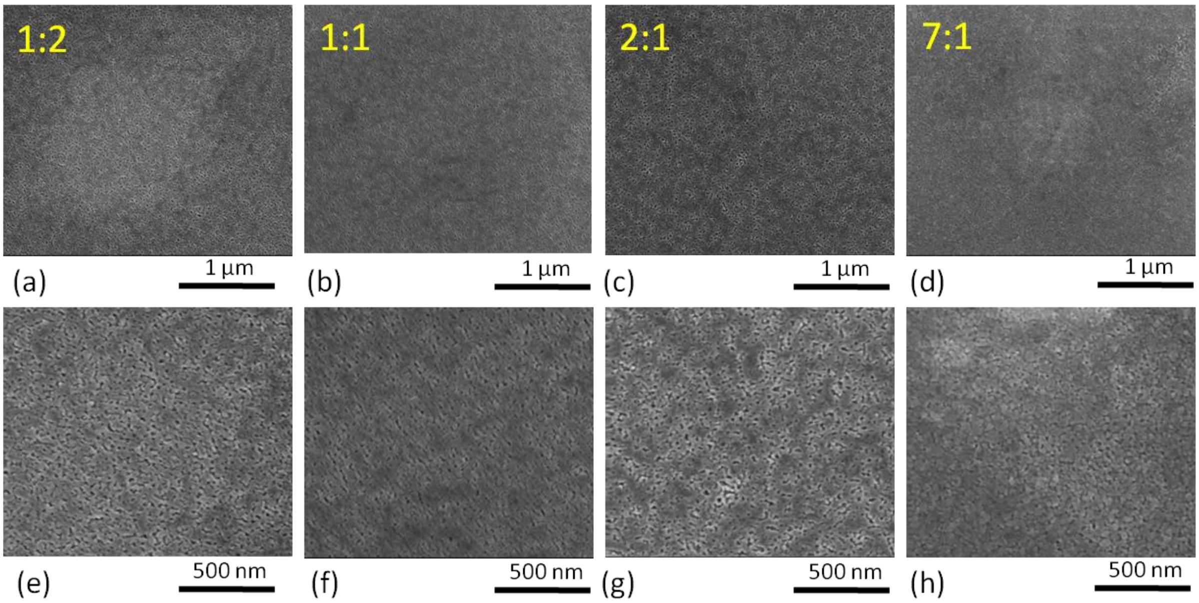

Figure S2. SEM images of β-Ga_2O_3 thin films prepared with different EtOH to water ratios: (a) and (e) 1:2, (b) and (f) 1:1, (c) and (g) 2:1, (d) and (h) 7:1.

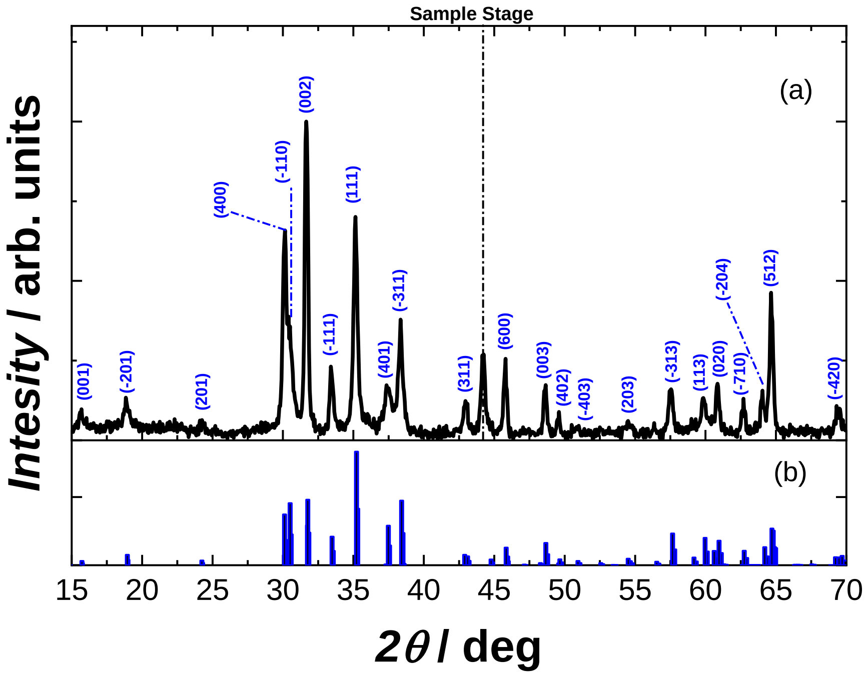

Figure S3. a) Indexing of the XRD pattern. Sample - flake microstructures. Assignment to β-Ga_2O_3 according to the ICSD card #83645; b) XRD pattern simulated using VESTA software (ref. [63] in the main text) and β-Ga_2O_3 crystal structure from the Crystallography Open Database (COD), card number #2004987.

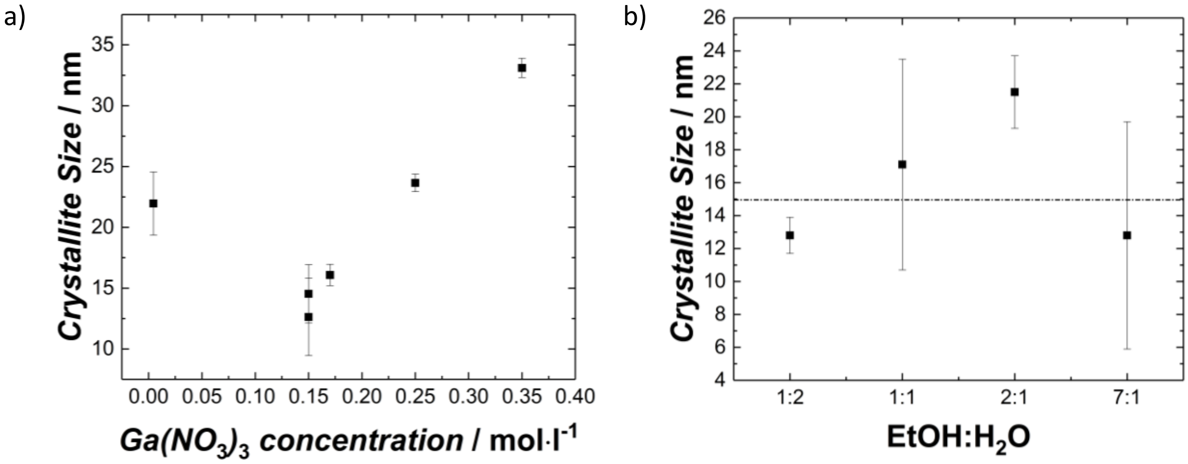

Figure S4. a) Calculated crystallite size for β-Ga_2O_3 microstructures in dependence of the $Ga(NO_3)_3$ concentration; b) Calculated crystallite size for β-Ga_2O_3 thin films obtained with different EtOH:H_2O ratios.

Table S1. Explanation for the origin of the Raman modes. Mode position is taken from the Raman modes of the thin β-Ga_2O_3 films. Notation of the atoms is adopted from refs [41,57] cited in the main text.

Mode position	Symmetry	Example of corresponding atoms
200.3	A_g	Libration and translation of chains
319.8	A_g	Torsion around Ga_1-O_7 axis
342.9	A_g	Bending of O_{11}-Ga_1-O_9 & O_5-Ga_1-O_7
349.7	B_g	Bending of O_5-Ga_1-O_9
415.9	A_g	Bending of O_5-Ga_1-O_7 & Stretching of O_{11}-Ga_1-O_9
475.0	A_g	Bending of O_{13}-Ga_3-O_{11} & Stretching of Ga_1-O_5
628.3	A_g	Stretching of Ga_3-O_9
651.6	B_g	Stretching of Ga_1-O_7
658.6	A_g	Stretching of Ga_3-O_{13} & Bending of O_{13}-Ga_3-O_{11}
765.6	A_g	Stretching of Ga_1-O_5 & Ga_3-O_{13}

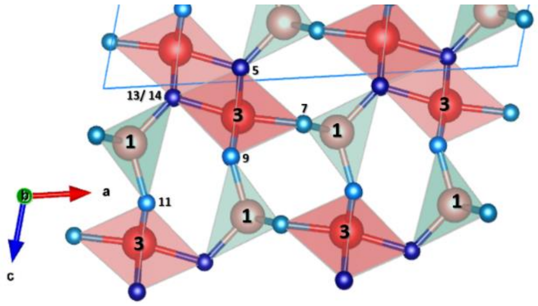

Figure S5. Sketch of the crystal lattice of β-Ga_2O_3 (created with VESTA software, ref. [63] in the main text) with indication of Ga and O atoms for the explanation of the origin of the Raman modes.

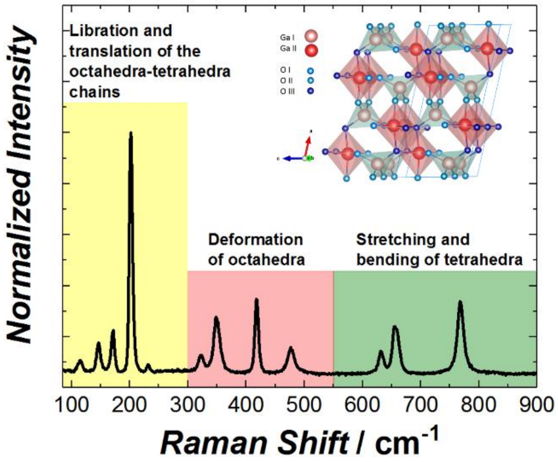

Figure S6. Raman spectrum of bulk like β-Ga_2O_3 on quartz glass taken with an excitation of 514.7 nm separated in the three parts of the spectrum.

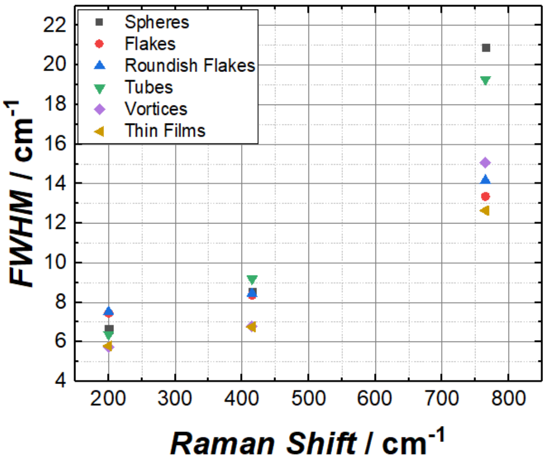

Figure S7. FWHM of the Raman modes for different β-Ga_2O_3 microstructures. For every part of the Raman spectrum of β-Ga_2O_3 we chose one mode (200 cm^{-1}, 415 cm^{-1}, and 765 cm^{-1}).

Table S2. Stress and Raman shifts for the different microstructures.

Microstructure	Stress / GPa	Experimental Raman shift / ± 0.6 cm^{-1}	Experimental Raman shift / ± 0.6 cm^{-1}	Experimental Raman shift / ± 0.6 cm^{-1}
Flakes	-1.68 ± 0.31	199.3	415.3	764.9
Round flakes	-3.15 ± 0.63	200.1	416.0	766.0
Tubes	1.68 ± 0.52	199.2	414.9	759.6
Vortices	2.00 ± 0.31	200.2	415.9	765.5
Spheres	-5.98 ± 0.31	199.7	415.6	763.5

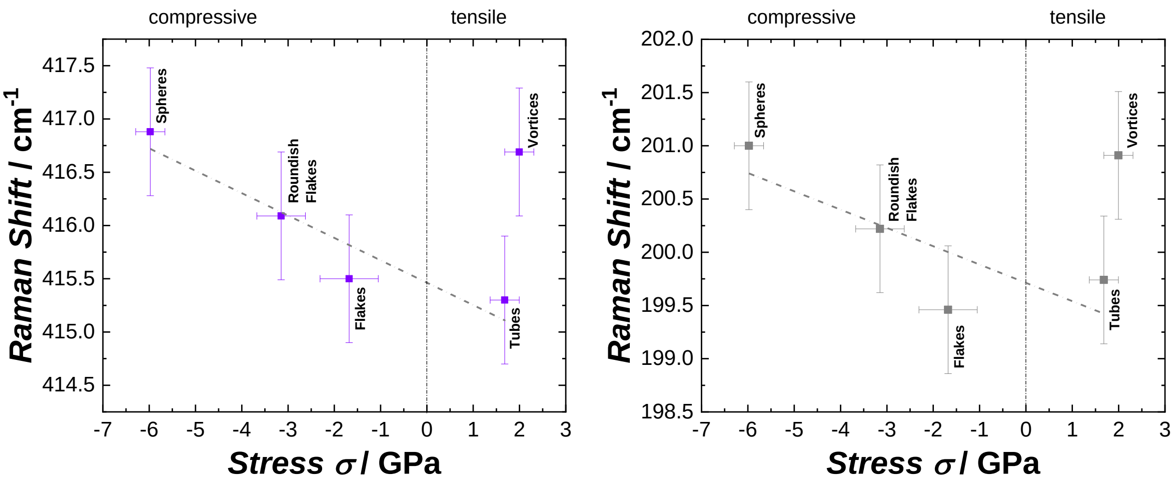

Figure S8. Raman shifts of all microstructures in dependence of the measured stress: a) mode around 200 cm^{-1}, b) mode around 415 cm^{-1}. In every case a negative slope from compressive to tensile stress is visible. The dashed line is an guide for the eye. Only vortices do not fit to the observed trend.

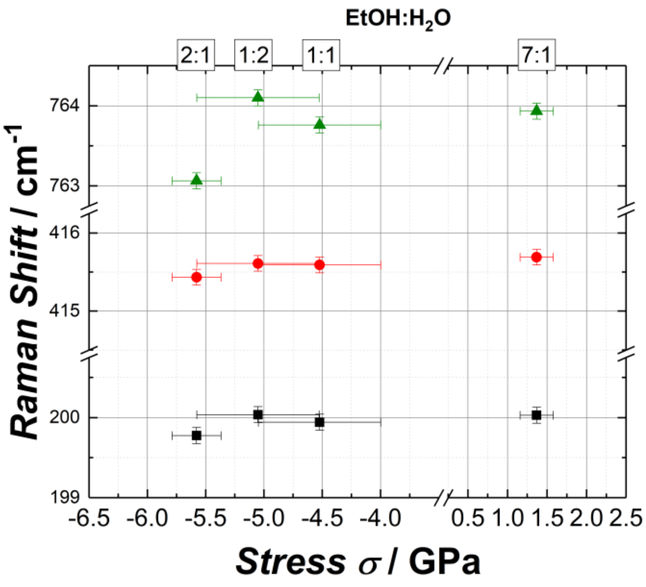

Figure S9. Raman shifts of thin films prepared at different EtOH:H_2O ratios in dependence of the measured stress for the modes around 200 cm^{-1}, 415 cm^{-1}, and 765 cm^{-1}. While the Raman shift is almost constant, the stress changes from compressive to tensile by adding more ethanol to the solution.

References (the same references are cited in the main text):

41. Schmidt, C.; Rahaman, M.; Zahn, D.R.T. Conversion of 2-dimensional GaSe to 2-dimensional β-Ga_2O_3 by thermal oxidation. *Nanotechnology* **2022**, *33*, 045702. https://doi.org/10.1088/1361-6528/ac2f5d.

57. Dohy, D.; Lucazeau, G.; Revcolevschi, A. Raman spectra and valence force field of single-crystalline β Ga_2O_3. *J. Solid State Chem.* **1982**, *45*, 180–192. https://doi.org/10.1016/0022-4596(82)90274-2.

63. Momma, K.; Izumi, F. VESTA 3 for three-dimensional visualization of crystal, volumetric and morphology data. *J. Appl. Crystallogr.* **2011**, *44*, 1272–1276. https://doi.org/10.1107/S0021889811038970.
